# Experiences of Adults With Type 1 Diabetes Using Digital Health Technology for Diabetes Self-Care: Qualitative Study

**DOI:** 10.2196/79704

**Published:** 2026-03-26

**Authors:** Divya Anna Stephen, Jan Nilsson, Unn-Britt Johansson, Anna Nordin

**Affiliations:** 1 Department of Health Science, Faculty for Health, Nature and Technology, Karlstad University, Universitetsgatan 2, Karlstad, 651 88, Sweden, 46 733016592; 2 Section for Advanced Nursing, Faculty of Social and Health Science, University of Inland Norway, Elverum, Norway; 3 Department of Health Promoting Science, Sophiahemmet University, Stockholm, Sweden

**Keywords:** digital technology, experiences, qualitative research, self care, type 1 diabetes mellitus

## Abstract

**Background:**

Type 1 diabetes is a constraining disease due to the burden of its management, and diabetes outcome largely depends on the effectiveness of diabetes self-care. Digital health technology (DHT), which includes continuous glucose monitoring, insulin delivery devices, and related mobile health apps, can support diabetes self-care and thereby improve diabetes outcomes. In literature, experiences with the use of DHT vary widely among people with diabetes and are a less studied area among adults with type 1 diabetes.

**Objective:**

The study aimed to explore experiences of using DHT for diabetes self-care among adults with type 1 diabetes.

**Methods:**

A qualitative design with an inductive approach was used. Adults with type 1 diabetes who are users of DHT and could understand Swedish were included in the study. Participants were recruited primarily via digital advertisements through social media. A convenient sampling method was used. Data were collected through open-ended questions in a web-based survey (autumn 2022) and 2 digital group interviews (autumn 2024). The survey questionnaire and interview guide attempted to capture positive and negative experiences of using DHTs for diabetes self-care through personally relevant incidents and behavioral details. Data from a total of 161 participants (n=156 survey participants and n=5 interview participants), using 1 or more forms of DHTs, were included in the study. Data were analyzed using qualitative content analysis with an inductive approach as per Graneheim and Lundman. The data in this study generated 324 meaning units relevant to the aim.

**Results:**

The participants experienced using DHTs in diabetes self-care as a balancing act between feeling empowered and feeling exasperated. This is described under 5 categories: promoting autonomy in daily life, self-awareness through collaborative learning, feeling secure, tackling technical challenges and the need for support, and navigating the burden of psychosocial challenges. DHTs were experienced as empowering when they supported autonomy in daily life, enhanced self-awareness through collaborative learning, and fostered a sense of security. However, having to tackle technical challenges and the need for support, and navigating the burden of psychosocial challenges, led to feelings of exasperation. The exasperating experiences hindered participants from experiencing a full sense of empowerment with DHT use.

**Conclusions:**

This study sheds light on both positive and negative experiences of using DHTs for diabetes self-care in a real-life setting. The exasperating experiences may widen the digital health inequities and therefore are important to address. Improving technological literacy and ongoing support from health care or device manufacturers may help users to address exasperating experiences. Further studies are needed to validate our findings.

## Introduction

Type 1 diabetes places a unique burden of management on the individual. It is an autoimmune disorder leading to severe endogenous insulin deficiency [1]. It requires the individual to be always in control of the disease. The latter can be experienced as constraining life [2]. The incidence and prevalence of type 1 diabetes has increased globally [3] with Sweden having one of the highest age-standardized incidences both among children [4] and adults [5]. Effective diabetes self-care plays a role in determining the outcome of diabetes treatment [1]. The 7 recommended diabetes self-care behaviors are healthy coping, healthy eating, being active, taking medication, monitoring, reducing risk, and problem solving [6]. These self-care behaviors and the interplay between them affect glycemic control and occurrence of complications [7]. However, despite the relentless self-care measures, people still experience loss of control due to their inability to address unforeseen factors that impact glucose levels [2]. New approaches and devices should be incorporated into diabetes care to minimize the burden of living with type 1 diabetes [1]. Newer technologies help to synthesize information into an easily understandable and interpretable form, enabling its application to self-care. They can engage, encourage, and motivate people with diabetes to manage their self-care [6].

Digital health technology (DHT)—or diabetes technology—includes continuous glucose monitoring (CGM), insulin delivery devices, and related mobile health (mHealth) apps. Insulin delivery devices include continuous subcutaneous insulin infusion (CSII), automated insulin delivery (AID) systems, and smart or connected insulin pens [8,9]. The DHTs have been found to improve glucose outcomes in people with diabetes [8,10]. In addition, the data generated by them is perceived to make life easy for people with type 1 diabetes [11]. Various forms of insulin delivery devices and CGMs have been available to people with type 1 diabetes from the late 1970s [12] and 1999 [13] respectively. However, after the 2010s, the technological developments have continuously brought new features and functionalities [12,13]. Although this development potentially makes diabetes self-care easier, it may also act as a barrier to effective uptake [8]. Some commonly reported barriers and burdens to DHT use were cost, numerous alarms, discomfort, need for engagement, and workload [14].

Experiences offer important information about the nature of people’s needs pertaining to various interventions [15], including DHTs. DHTs such as CGMs [16,17], CSII [18], and AIDs [19,20] were experienced as tools to help reduce glucose variability, improve glucose control [17,19,20], and overall diabetes burden [20], offering people with diabetes more control over their own lives [16-18]. The DHTs were experienced as convenient, enhancing self-care engagement, and providing confidence [16,17]. However, DHTs can place a significant burden on people due to the need for continuous interaction with devices when it comes to placement, calibration, and troubleshooting [19,21], and data overload [18,21]. They also cause challenges for people by drawing unwanted attention [18,21], disruptive alarms and alerts causing disturbance to others as well as to daily life [16,19-21], body image issues, and issues with devices during sports [21]. Other challenges were physical discomfort [16,18] due to their invasive nature, and skin reactions [21]. Additionally, unfamiliarity with technology, malfunctioning and inaccuracy of devices, mistrust in algorithms, economic constraints, supply deficit [16], sensor problems, not matching people’s expectations [19], and uneasiness in relinquishing control to the system [19,20] were also reported challenges. The challenges with DHTs can be experienced as a burden [21]. Thus, experience with the use of DHT varies widely among people with diabetes.

DHTs have evolved rapidly, which has had a positive impact on diabetes self-care and glucose outcomes. However, people’s expectations, the current state of technology, as well as access to technical support and health care support may differ in a real-world setting compared to randomized controlled trials. Therefore, it is essential to explore people’s experiences of using DHTs in day-to-day life. This knowledge may contribute toward understanding and improving the gaps that prevent effective use of DHTs in diabetes self-care. Nevertheless, studies in this area in a long-term, real-world setting are rare. In Sweden, the DHTs are available to people with type 1 diabetes through a publicly funded high-cost protection scheme [22,23] and therefore usage rates are high [24,25]. This provides a unique opportunity to explore experiences among long-term users. Thus, the aim of this study was to explore experiences of using DHT for diabetes self-care among adults with type 1 diabetes.

## Methods

### Study Design

The study had a qualitative design with an inductive approach and was reported as per consolidated criteria for reporting qualitative research (COREQ) guidelines [26]. It includes data from 2 open-ended questions from a web-based survey and 2 group interviews to describe experiences of using DHT for type 1 diabetes self-care.

### Participants

Adults (18 years or older) with type 1 diabetes who were users of DHT and could understand Swedish were included in the study. Participants were chosen by convenience sampling method. A total of 162 participants answered the open-ended questions in the survey. However, data from 6 (3.7%) participants were excluded, as they did not describe an experience. This led to open-ended question data from 156 (96.3%) participants being included in the study. For the group interview, 12 participants had registered interest and were invited to participate. However, 7 (58.3%) participants could not participate for personal and technical reasons, resulting in 5 (41.7%) interview participants. Thus, a total of 161 participants provided data to this study. Included participants used 1 or more forms of DHT. Brands of DHT and mHealth app features used by participants are available in Table S1 and Table S2 in Multimedia Appendix 1.

### Data Collection

#### Survey

Participants were recruited between September and November 2022, primarily through digital advertisements on social media and completed a web-based survey (Survey&Report by Artisans media). Additionally, study advertisements were done at associations for people with diabetes in Sweden and a regional hospital. The survey included screening questions on age, diabetes type, and pregnancy status to determine participants’ eligibility. The survey automatically closed if any of the exclusion criteria (age <18 years, diabetes type 2, or pregnancy) were met. More information on the survey recruitment strategy is available in a previously published paper [25]. Two open-ended questions in a web-based survey, which was part of a larger study [25,27], provided data to this paper. Although paper surveys were available, their uptake was minimal, and none of the respondents provided answers to the open-ended questions.

The phrasing of the interview questions was inspired by the critical incident method [28,29]. The questionnaire began by asking participants to describe an experience where they were *satisfied* with their use of the mHealth app for diabetes self-care, and then to describe an experience where they were *dissatisfied*. They were then asked to relate details about the particular incident or situation and describe how they managed it, how it ended, and what they learned. Phrasing the questions this way helped participants recall and describe their positive and negative experiences using DHTs for diabetes self-care through personally relevant incidents and behavioral details [30]. The questions were face-validated among 9 adults with type 1 diabetes and 4 diabetes nurses, where after minor changes were made.

#### Group Interviews

An initial review of the web-based survey data was conducted by the first (DAS) and last (AN) authors to assess the need for further enrichment and updates, which was deemed necessary. Thus, 2 group interviews were conducted between August and September 2024. The participants for the group interview were recruited via digital advertisements through Facebook and Instagram. The interested participants could leave their informed consent and contact details through a digital link (Survey&Report by Artisans media) in the advertisement. The group interviews included 2 and 3 participants each. The participants were sent the interview invitation through email. The video conferencing tool Zoom Workplace was used to conduct the interviews. The end-to-end encryption feature in Zoom was activated to secure the room and ensure data privacy.

A semistructured interview guide, developed based on the open-ended questions in the survey study, was used for the interview. The questions were phrased slightly differently based on the data from the survey in order to include digital devices along with mHealth apps. Digital devices were added as survey answers showed that participants could not differentiate the functions of mHealth apps from those of their digital devices. The interview began in a similar way to the survey questionnaire, with an overarching question asking them to describe experiences of using mHealth apps and digital technology to manage type 1 diabetes. Similar to the questionnaire, this was followed by sub-questions. Follow-up questions were asked as needed: Can you tell me more? Can you elaborate on that? AN (PhD in nursing science and associate professor) was the main interviewer and has previous experience of conducting qualitative interviews and knowledge in the field of digital health innovations. DAS, a PhD student, acted as the observer and note taker. Both the interviewers were female, registered nurses and had no previous relationship with the participants. The interviews, which included only the interviewers and participants, lasted between 60 to 90 minutes and were audio recorded using a separate dictaphone. The audio recordings were transcribed verbatim by a transcription agency, which had a data processing agreement with Karlstad University. The interview transcripts were not returned to the participants for correction or comments.

### Data Analysis

Qualitative content analysis as per Graneheim and Lundman [31] was used to analyze the data. An inductive or data-driven approach characterized by the search for patterns was used [32]. AN and DAS initially reviewed survey data to determine the necessity for further data collection. However, no coding or in-depth data analysis was done on the survey data until the end of data collection through group interview. DAS and AN manually performed the initial coding of all data in Microsoft Excel using a convergent design, where both survey and interview texts were coded from scratch. Any disagreements were discussed among all 4 authors until consensus was reached. No qualitative data analysis software was used.

The survey text was read repeatedly and answers that described whole or part of an experience were assigned as a meaning unit by DAS. The meaning units were then condensed and coded close to the text by DAS and AN. Similarly, the interview text was read as a whole several times by DAS before extracting meaning units. It was then condensed and coded close to text by DAS and AN. The codes were then grouped based on their similarities and differences into 5 categories and a theme. Data saturation was discussed post hoc and was deemed as achieved when categories were found repeating [33]. A back-and-forth process of going between data, codes, and categories was carried out to ensure codes fit the category. The categories and themes were discussed among all 4 others until consensus was achieved. The data in this study gave rise to 324 meaning units with relevance to the aim, of which one-third were from group interviews [31,32]. The coding and the categorization were discussed several times in the research group and at the research seminars at the research school to strengthen confirmability and credibility. All 3 authors except the first author were experienced in qualitative research and guided the first author through the process. See Table 1 here and Table S3 in Multimedia Appendix 2 for examples of the data analysis process. Participants were not consulted for feedback on the findings.

**Table 1.** Example of the data analysis process.^a^

Meaning unit	Condensed meaning unit	Code	Subcategory	Category
*I have always had problems with low blood sugar at night...usually because I exercise later in the evening. I am very happy that my device wakes me up at night when my blood sugar is low so that nothing serious happens and next day is not ruined by consequences of my low blood sugar at night*. [P283, 34 y, female, using CGM^b^]	Waking me up at night with low blood sugar, preventing complications as well as the next day being subsequently ruined	Low blood sugar alarms at night prevent complications and the next day being ruined	Alarms and warnings prompt timely intervention	Promoting autonomy in daily life
*Because it’s no fun sitting in a court that’s like a library...And then comes that beep and then the phone rings five seconds right after. Not the right context...I solved it by looking terribly embarrassed and then in the break I bolted (laughter). It’s like maybe a situation where you want to close your eyes*.[P452, 46 y, male, using CGM and CSII^c^]	Alarm from device and later from phone during a session at court led to feeling embarrassed and stressed	Alarms at inconvenient places are embarrassing and stressful	Source of social awkwardness	Navigating the burden of psychosocial challenges

a Theme: A balancing act between feeling empowered and feeling exasperated.

b CGM: continuous glucose monitoring.

c CSII: continuous subcutaneous insulin infusion.

### Ethical Considerations

This study follows the ethical guidelines as per the Helsinki declaration [34]. Participation in the study was voluntary, and no compensation was provided. Participants were given oral and written information about the study and their right to withdraw participation at any time. Informed consent was obtained from the participants digitally via the survey tool. The data related to this study were stored on Sunet Drive, a secure cloud storage service, and on an appropriately encrypted USB drive kept in a locked archive at Karlstad University, accessible only to the research team. In accordance with institutional guidelines, the data will be retained at Karlstad University for a period of 10 years. The Swedish ethical review authority approved the study before data collection commenced (Dnr: 2021-05337-01, Dnr: 2022-04079-02, Dnr 2024-02691-02).

## Results

In total, 161 participants provided data for this study. The participant characteristics are summarized in Table 2.

**Table 2.** Characteristics of participants who answered the open-ended question in the survey and participated in the focus group interviews.

Characteristics	Values	
	Survey (n=156)	Interview (n=5)
Age (y), median (range)	36.5 (18-79)	48 (25‐68)
Duration of diabetes (y), median (range)	18 (<1‐72)	5 (1-43)
Duration of digital health device usage (y), median (range)	—^a^	5 (1-8)
Gender, n (%)		
Women	104 (66.7)	2 (40)
Men	52 (33.3)	3 (60)
Education level, n (%)		
University level education	93 (59.6)	—
Primary/secondary school	63 (40.4)	—
Digital devices used^b^, n (%)		
Blood glucose monitor	79 (50.6)	5 (100)
Continuous glucose monitoring	144 (92.3)	4 (80)
Continuous subcutaneous insulin infusion	45 (28.8)	1(20)
Automated insulin delivery systems	42 (26.9)	1 (20)
Smart insulin pens	19 (12.2)	—
Mobile health apps	156 (100)	5 (100)

a Not available.

b Participants could use more than one digital device.

### Participants’ Experiences of Using DHTs

#### Theme: A Balancing Act Between Feeling Empowered and Feeling Exasperated

The participants experienced using DHTs in diabetes self-care as a balancing act between feeling empowered and feeling exasperated. This is described under five categories: promoting autonomy in daily life, self-awareness through collaborative learning, feeling secure, tackling technical challenges and need for support, and navigating the burden of psychosocial challenges. DHTs were experienced as empowering as they helped people take control over their lives by promoting autonomy in daily life, self-awareness through collaborative learning, and making them feel secure. However, participants have also described having to tackle technical challenges and the need for support and navigating the burden of psychosocial challenges, which left them feeling exasperated. Thus, participants conveyed an experience of having to balance between feeling empowered and feeling exasperated. The exasperating experiences prevented them from experiencing a full sense of empowerment with DHT use. Textbox 1 depicts the various categories and Table 3 depicts subcategories and count of codes within each category.

Textbox 1. **Summary of participants’ experiences of using digital health technologies for self-care in type 1 diabetes.**

A balancing act between feeling empowered and feeling exasperated includes the following categories:

- Tackling technical challenges and the need for support
- Promoting autonomy in daily life
- Self-awareness through collaborative learning
- Navigating the burden of psychosocial challenges
- Feeling secure

**Table 3.** Subcategories and count of codes within each category (n=324).

Categories and subcategories	Count of codes, n
Tackling technical challenges and the need for support	145
Hassles with DHT^a^ malfunction and usability	
Glucose value inaccuracies	
Unreliable alarms and warnings	
Health care support need	
Promoting autonomy in daily life	108
Aiding glycemic control	
Alarms and warnings prompt timely intervention	
Eases insulin dosing	
Self-awareness through collaborative learning	28
Understanding factors affecting glucose variability	
Implementing self-care changes based on value assessment	
Eases discussion on diabetes self-care	
Navigating the burden of psychosocial challenges	27
Psychological impact and response	
Source of social awkwardness	
Feeling secure	16
Experiencing stability in their lives	
Safety and control over their disease	

a DHT: digital health technology.

#### Category 1: Tackling Technical Challenges and the Need for Support

A large number of participants with type 1 diabetes described their experiences of tackling technical challenges and need for support. They described hassles with DHT malfunction and usability, glucose value inaccuracies, unreliable alarms and warnings, and health care support need. Tackling technical challenges with DHT and the need for support led to exasperation.

DHT malfunctions experienced by participants ranged from software-related issues such as failure in updating values and display inaccuracies to device dysfunctions. Unreliability in alarms and warning systems was also described, including false alarms and failing to alert due to either DHT malfunctions or design flaws. Alarms were also experienced as developmentally immature when they could not be personalized or overridden. Identified causes of DHT malfunction described by the participants were either related to sensor issues, dysfunctions when approaching end of lifespan of device parts, incompatibility with extreme external temperatures, physiologic changes (eg, infection) or diet causing rapid glucose fluctuations, device connectivity issues with mHealth apps or other connected devices, lack of device compatibility with other devices or newer software, and usability issues (eg, navigational difficulties and limited personalization options).

*I also noticed that this summer when it was like 30 degrees in the sun and you sat outside, then..for me the values went upwards. It looked as if I had tucked in myself with 1 kg sugar. And then you come in and it takes five minutes and it looks like it is reversing down but it stays stable,...it was really something that you learned that one needs to adjust for both heat and cold...it should be just moderate all the time*.

[P455, 25 y, male, using CGM]

Participants experienced disruptions in connected device functions (eg, AID system), lack of access to glucose values, and need for reliance on nonapproved apps due to technical challenges. In addition, participants reported technical challenges as contributing to a cascade of poor self-care decisions—beginning with uncertainty around glucose values and insulin dosage estimation, which led to incorrect dosing, potentially resulting in compensable or noncompensable hyperglycemia or hypoglycemia, and in some cases, the need for emergency care. Participants described eventually learning to navigate the technical challenges by using DHTs as a guide and relying on their own body symptoms and sensations for accurate feedback. When a mismatch in DHT information and bodily symptoms was noticed by participants, it was counterchecked with glucose graphs or with blood glucose monitors. Some malfunctions required the participants to correct underlying causes such as relieving pressure on devices and change of sensor or parts of insulin delivery devices. The latter, however, was reported by the participants as requiring backup material, being uncomfortable if in public eye, irritating to the skin, and required waiting out the “warm up” period. To keep using the DHTs, participants narrated the need for relentlessly being in control through careful organization and preparation. Even then, the DHTs could still fail, causing disruptions to daily routines. DHTs were described as “a shackle” when preventing spontaneous activities due to a lot of backup need.

*But it’s a big difference in some way, I feel a little more shackled and a little more exposed when I'm dependent on the [insulin] pump...with the pump there is so much, there are infusion sets, there are needles, there is the insulin, it has to be cold and...you have to charge it...There it can very easily tip over and become stressful instead.*

[P452, 46 y, male, using CGM and CSII]

Participants felt that these technical challenges induced value speculations, counterchecks, wrong self-care interventions, and subsequent correctional measures that were unnecessary and inconvenient, disrupting routines, stressful, tiresome, and undermining trust in DHTs. The absence of prompt technical assistance was described by participants as a factor contributing to disengagement and eventual discontinuation of the DHT. Some participants shared that they received minimal support from healthcare for DHT use, often turning to social media for help. Technical challenges frequently occurred outside of health care providers’ consultation hours, highlighting a lack of around-the-clock support. Using DHTs with minimal health care support was compared by participants to that of a “technical sport—a sport requiring knowledge of technology, extensive exploration and adaptation without which you lag in the race.” Participants expressed a need for support in better understanding the technology and selecting the most suitable option from those available. Participants felt that adequate support would prevent them from feeling chained to ill-fitting DHTs. Some found it helpful to have consistent contact with the health care personnel who recommended the DHTs. However, participants voiced that this level of support requires health care professionals to maintain up-to-date knowledge of available DHTs. Participants also described a lack of knowledge among health care personnel in primary care and home health care when it came to caring for people with type 1 diabetes using DHTs.

*And the thing is also that nothing with technology or with diabetes happens when the diabetes nurse has opening hours between 8 and 9 in the morning...but it usually occurs in the evenings...you sometimes forget a little human and as I said, it takes quite a lot and I feel...if you do not really have that interest [yourself]...you easily...fall behind in it.*

[P454, 48 y, female, using CGM]

#### Category 2: Promoting Autonomy in Daily Life

Participants with type 1 diabetes felt that DHTs promoted autonomy in daily life by aiding glycemic control, easing insulin dosing, and prompting timely intervention through alarms and warnings. Participants described this as being achieved through frequent glucose checks and tracking, decision support to intervene in time through alerts, alarms, and warnings, and assisting with insulin administration in everyday life as well as during special situations (eg, infection or while having a multicourse meal). In comparison to a regular finger prick test with blood glucose monitor, participants felt that DHTs promoted autonomy by making self-care monitoring easier, frequent, discreet, pain-free, and always accessible. Through graphs, trends, and alarms, participants described receiving a comprehensive and uninterrupted overview of glucose values or insulin dose, which was unattainable through occasional finger prick tests or glycated hemoglobin (HbA_1c_) tests. This comprehensive and uninterrupted diabetes monitoring and management was identified by participants as helpful to keep glucose values in normal range and improve “time in range.” Participants mentioned this as reducing worry of hypo- or hyperglycemia while being engaged in various activities or work and at night. Therefore, DHTs were experienced as a lifeline working around the clock, preventing complications or disruptions to daily routines. By giving enough time to intervene to correct glucose levels, they provided participants with the freedom of activity. Participants described that DHTs enabled them to live their lives without having to think too much about their disease.

*I think Pump...it’s really great, both accelerates and brakes, it helps to go down at night...then it stops the insulin needed...[when] you're almost low so it...starts warning with four...it manages the night...but otherwise...I would have been woken up in the night...or something else could have happened. So that’s the advantage.*

[P453, 68 y, male, using AID]

*Every time it alerts for the blood sugar...high value over 11 mmol...I have time to correct before it gets higher. Thanks to this...HbA1c went from 70 to 54 in about 1 year*.

[P204, 26 y, female, using CGM and CSII]

#### Category 3: Self-Awareness Through Collaborative Learning

Participants with type 1 diabetes voiced that DHTs contributed to a greater self-awareness about their disease and the self-care strategies required. Achievement of this increased self-awareness was described by participants as being facilitated by participants’ collaborative engagement and learning with DHTs. The participants described this experience under the subcategories: understanding factors affecting glucose variability, implementing self-care changes based on value assessment, and easing discussion on diabetes self-care with health care personnel.

DHTs were experienced as helping participants learn about diabetes and the impact on glucose levels of various factors such as exercise, insulin dosing, stress, menstrual cycle, and food, thereby facilitating self-care. Participants described achieving this learning through the DHTs’ graphical display and trends, which help find patterns, recurring events, habits, and reason for deviant values. The insight gained helped participants make self-care changes and prevent glucose variability. Alarms and predictions helped them learn differences in body symptoms with deviant glucose values. In contrast to periodic finger prick testing using blood glucose monitors, DHTs helped users learn to time the effects of insulin and food. By aiding participants with analyzing and evaluating the interplay between various factors influencing glucose levels, DHTs helped lower HbA_1c_. Participants also felt that the uploaded or shared data from the DHTs made it easier to discuss diabetes self-care with health care personnel even without in-person visits. Graphs from devices were reported to act as a discussion tool in health care meetings.

*By continuously reviewing graphs, I was able to see patterns in my daily curve and deduce my high blood sugar readings to previously low readings due to low food intake/lack of snacks. Through this, I was able to make adequate adjustments to reduce the occurrence of both low and high blood sugar values and ensure a more stable curve.*

[P388, 30 y, female, using AID]

*I exercise a lot...demanding work and comes up with spontaneous things. With CGM and connection to watch and alarm, I have enough knowledge for good planning...I have above all learned how to plan exercise and insulin / food before and during sessions that can last many hours. Then how these affect my long-acting insulin, which must be lowered by up to 50% after intensive exercise. Decent control of the curves...along with the motivation to exercise.*

[P63, 48 y, male, using CGM]

#### Category 4: Navigating the Burden of Psychosocial Challenges

Experiences of psychosocial challenges while using DHTs were also described by participants with type 1 diabetes, under subcategories psychological impact and response to DHTs and a source of social awkwardness. Participants described feeling compelled to maintain values within the target range with DHT use, making them fixated on achieving the perfect “time in range” values and overly trusting DHTs above bodily symptoms. Participants felt that this led to stress and a sense of self-competition, particularly during the initial phase of technology use. Over time, some participants learned to ease this pressure and place less importance on staying within the perfect range. Such trust in DHTs above bodily symptoms caused unnecessary anxiety in participants about DHTs failing to work or when seeing false “in range” percentages. The obsession to remain in target range and high focus on DHTs was also described by participants as leading to overcorrection, unnecessary interventions, incorrect insulin dosing, fluctuating values, and unwanted consequences. Additionally, it could lead to values, alarms, and reminders taking over every part of participants’ life. DHTs were thus experienced as disturbing peace and quiet, causing enormous stress and frustration, which eventually led to technology fatigue. Taking a day off DHTs helped some participants realize that they are no more than aids to diabetes self-care.

*Sometimes I can get very obsessive and frustrated when I'm high and the curve doesn't want to turn as quickly as I expected, I can be tempted to take more doses of the insulin. When the curve then turns, it goes too fast and I have to correct with extra carbohydrates.*

[P252, 56 y, female, using CGM]

*It gives a huge stress to follow glucose levels in the app all the time along with the warning/alert sounds day and night when blood sugar sways. One does not get any peace and quiet. This gives an enormous stress along with other things one should achieve/tread through in life.*

[P16, 49 y, female, using CGM and smart insulin pen]

DHT can also cause social hassles and awkwardness. Participants described frustration over uneducated comments from others about DHTs as well as 1-sided (ie, only the positives) demonstrations of DHTs on social media. Another problem described was that different counties offer different DHTs, making it difficult to move to a different county after choosing a certain DHT. Placement and access of DHT while in public places or events were also experienced as challenging due to physical exposure. One participant felt undignified when people showed aversion and judgment toward DHT alarms. Lack of customization like snoozing, overriding, or volume adjustments for alarms was described by participants as causing embarrassment, disturbance in public places, and in extreme cases, discontinuation of DHT.

#### Category 5: Feeling Secure

Participants with type 1 diabetes experienced feeling secure when using DHT for diabetes self-care. They described this under experiencing stability in their lives and safety and control over their disease with DHT use. Participants reported a sense of safety since alarms would alert them to deviant glucose values in work-related meetings and at night when asleep. This, in turn, gave them a feeling of being in control, stability, and reduced stress about complications such as diabetes-related coma. Some participants described how the follower function saved their lives by alerting their relatives to intervene in the event of low blood sugar, which went unnoticed by the participants.

*The follower function where my daughters helped me with too low blood sugar to avoid going into a [diabetes-related] coma! I did not alert myself...about the low blood sugar, or I became low too fast and could not react. I...don't feel sensations anymore, until the blood sugar is too low.*

[P382, 79 y, male, using CGM and smart insulin pen]

*Alarms about low glucose values at night have helped to reduce stress about dangerous situations occurring when I am not aware.*

[P305, 22 y, male, using CGM]

## Discussion

### Principal Results

This study examines adults’ experiences of using DHT for diabetes self-care in real-life settings. To our knowledge, research in this area among adults with type 1 diabetes remains limited. The findings reveal that these experiences reflect a balancing act between feeling empowered and feeling exasperated, discussed under the following categories: promoting autonomy in daily life, self-awareness through collaborative learning, feeling secure, tackling technical challenges and need for support, and navigating the burden of psychosocial challenges. In line with our findings, use of DHTs [35], CGM [36], and AID [37] has been reported as empowering people with type 1 diabetes [36,37]. However, this study’s findings also indicate that participants were not able to fully feel empowered due to feelings of exasperation with DHTs. Another related study also found no association between the total diabetes empowerment score and the DHT factors among the same sample [25]. Therefore, larger studies exploring empowerment using well-established patient-reported outcome measures among this population are needed to validate our findings.

In this study, DHTs were experienced as promoting autonomy in daily life. Other studies have also reported findings in relation to this category, such as feeling liberated [17] and getting part of one’s life back [17], aiding glucose control [16-18] and reducing disease-related restriction on activities [38]. Participants experienced feeling secure in their daily lives and with their diabetes self-care when using DHTs, which is in line with findings from other studies among adults with type 1 diabetes [2,17]. Contrary to our results, 1 study reported participants’ reluctance to use CSII pumps due to a preference for the well-known, technology skepticism, and a belief that it would diminish their sense of security and control [39]. The follower function, in line with our findings, can foster a sense of safety [16] or can be a source of conflict when perceived as surveillance [36]. In our study, DHTs were experienced as increasing awareness of diabetes self-care through collaborative learning, which is similar to findings by Cleal et al [18].

Tackling technical challenges and the need for support is one of the largest categories in this study. Our findings are similar to findings from other studies on experiences of technical challenges like disturbing alarms [16,40], malfunctioning [16,40,41], and value inaccuracy [16]. Descriptions of some incidents falling in this category were challenges, which were known and clearly described by DHT manufacturers in their user manuals. Furthermore, navigating the burden of psychosocial challenges with DHTs is another finding consistent with reports from previous research [21]. Participants’ experiences within this category correspond to well-documented constructs like diabetes distress with DHT use [42], alarm fatigue [43], frustration with technology [21,44], and alarm embarrassment [44]. These findings indicate a need for structured periodic education and training on DHTs, as a way to help people with type 1 diabetes effectively tackle technical, emotional, and social challenges related to DHTs [8,21]. This also indicates the need for health care personnel or systems to address this educational need [8]. A continued education program trial for insulin pump users was found to improve the experimental groups’ glucose control [45]. Thus, improving people’s technological literacy is a way to address disparities in DHT usage [46]. Need for support was a finding in this study, which to our best knowledge was not reported elsewhere. Getting continued support for DHT use is essential in keeping up the morale of people with type 1 diabetes. More studies on the need for support while using DHTs are therefore warranted to validate these findings.

### Methodology Discussion and Limitations

Inference from participant data suggests that the study successfully recruited individuals from various regions of Sweden. This enabled the capture of some regional variation in the provision of DHT for individuals with type 1 diabetes [24]. However, collecting explicit information on participants’ regions would have ensured greater representativeness. The inclusion of participants of both genders, varying ages, diabetes durations, and educational backgrounds further enhances the transferability of the findings. In addition, the interview participants also varied in their duration of DHT use. However, data on the duration of DHT use were not collected in the survey, which could have further added to the information on transferability. The open-ended question data was pseudonymously collected by the survey tool. Data from interviews was pseudonymized using handwritten codes to ensure that no participant could be individually identified through any of their attributes in the published results. The interview participants were not connected to anyone in the research group. The 4 researchers involved in this study had differing clinical and qualitative experiences. The first and third authors have experience working with patients with type 1 diabetes. Previous studies done by the research group in this area give them a preunderstanding of how people with type 1 diabetes experience using DHTs. This preunderstanding can create a bias during data analysis, but this was minimized by the varied experiences of the other 2 authors, as well as discussions about data analysis in the research group and research seminars at the research school. The preunderstanding was also an advantage when it came to understanding the varied DHT terminology participants used during data collection.

The data for this study were obtained through open-ended questions in a digital survey conducted in autumn 2022 and 2 digital group interviews in autumn 2024. Initially, we aimed to conduct focus group interviews; however, due to the low number of participants, the level of interaction required to generate meaningful data in focus groups could not be ensured [47]. The survey contributed a large number of meaning units, which was a strength of this data source. Nevertheless, these meaning units varied considerably in length (10‐150 words) and richness. The interview guide, inspired by the critical incident technique, facilitated the capture of experiences from a sufficient number of participants. The inclusion of both positive and negative subquestions ensured that experiences were not described from a single perspective. Several survey responses lacked the outcome component (ie, self-care measures taken to manage the incident) required for inclusion as a critical incident, which is considered essential for credibility in studies using the critical incident technique [29,48]. Consequently, following the critical incident method throughout this study was not considered appropriate. The data were analyzed inductively; however, a deductive approach using a rigorous theoretical framework could have further enriched the findings.

By 2024, newer devices incorporating advanced algorithms had become available through the Swedish health care high-cost protection scheme, compared to 2022. The interviews carried out in autumn 2024 sought to explore potential changes in experiences with digital health technologies during this period. Nevertheless, given that only 5 participants were included, temporal variations in devices, algorithms, or reimbursement practices may not have been comprehensively captured. An added information on brands and models of each DHT could have provided more insights into participant experiences and enriched the study. Some data on digital technology brands, drawn from participant quotes, is available in Table S1 in Multimedia Appendix 1. However, this list of brands is not comprehensive, as it was not actively collected in the survey. Nevertheless, DHT will continue to evolve, while the categories derived from patient experiences are likely to remain relevant regardless of these changes. As technology advances, there may be variations in the intensity of patients’ experiences related to some of the categories. Therefore, the findings remain important despite the absence of detailed brand information on DHTs used by the participants.

Financial constraints affecting DHT uptake have been well documented in literature [16,49,50] and can contribute to diabetes distress, in turn affecting experiences of DHT use [21]. This aspect is not captured in this study results. This could be due to the high cost protection coverage extended to the DHTs by the Swedish public health care system [23]. This financial protection could have played a role in the high uptake of DHTs and shaping participants’ experiences reported in this study. Therefore, these results should be generalized with caution to settings with different reimbursement and support infrastructures. Participants were primarily recruited through social media, and data was collected digitally via survey and interviews. This approach enabled the inclusion of participants with diverse demographic backgrounds. However, the recruitment strategy introduces a risk of selection bias toward individuals who are digitally enthusiastic, potentially leading to the underrepresentation of nonusers, lapsed users, and older adults with limited digital literacy. Furthermore, individuals experiencing severe alarm fatigue who discontinued use, as well as those outside Sweden’s high-coverage digital context, are less visible in our data. Consequently, the findings of this study primarily reflect the perspectives of active and digitally engaged participants, and caution should be exercised when generalizing to populations with lower digital engagement or different infrastructural conditions. The self-reported diagnosis of type 1 diabetes through an online survey may be considered as another limitation of this study. However, 85% (133/156) of survey participants provided responses that included detailed descriptions of the diabetes technologies they use and how these influence their blood glucose or daily routines. In addition, 135 (86%) out of 156 survey participants reported being diagnosed before the age of 35 years, and 81 (52%) out of 156 before the age of 15 years, which together indicate a high likelihood that the self-reported diagnoses of type 1 diabetes are credible.

### Conclusions

The participants experienced the use of DHTs in diabetes self-care as a balancing act between feeling empowered and feeling exasperated. This study sheds light on both positive and negative experiences of using DHTs for diabetes self-care in a real-life setting. The exasperating experiences may widen the digital health inequities and are therefore important to address. Improving technological literacy and ongoing support from health care or device manufacturers may help users tackle technical challenges and navigate the burden of emotional and social hassles. Further research in this area—both within Sweden and internationally—is required to validate these findings and to facilitate their translation into clearly defined user needs and system-level interventions.

## Supplementary material

10.2196/79704 Multimedia Appendix 1 Brands and model of digital health technology and mobile health app features used by the participants.

10.2196/79704 Multimedia Appendix 2 More examples of the data analysis process.

## Abbreviations

AID: automated insulin delivery
CGM: continuous glucose monitoring
COREQ: consolidated criteria for reporting qualitative research
CSII: continuous subcutaneous insulin infusion
DHT: digital health technology
HbA_1c_: glycated hemoglobin
mHealth: mobile health
