# Supplementary material for: Experiences of Adults With Type 1 Diabetes Using Digital Health Technology for Diabetes Self-Care: Qualitative Study
Source: JMIR Diabetes. 2026 Mar 26;11:e79704. doi: 10.2196/79704 (PMC13021107; doi:10.2196/79704)
Supplement: Multimedia Appendix 1 [file diabetes-v11-e79704-s001.docx]

### Table S1: Brands and model of DHT used by participants in the study^a^

| **Type of DHT** | **Brand and model** |
| --- | --- |
| CGM^b^ | Freestyle libre 1 |
|  | Freestyle libre 2 |
|  | Freestyle libre 3 |
|  | Dexcom G6 |
|  | Eversense system |
| Insulin delivery systems | Dana Diabecare R insulin pump |
|  | Omnipod dash |
|  | Medtronic minimed 640G + guardian sensor |
|  | MiniMed 780g + guardian 4 sensor |
|  | DIY closed loop insulinpump |
|  | Tandem's t:slim X2 insulin pump with Control-IQ technology |
|  | Medtrum TouchCare® Nano System |
|  | Novopen echo Smart insulinpen |
| mHealth apps^c^ | Libre link and libreview |
|  | T1D app |
|  | Dexcom clarity, Dexcom G6 and Dexcom follow |
|  | Glooko |
|  | xDrip+ |
|  | Bubble Diabox |
|  | Android APS (AAPS) |
|  | Novopen plus |
|  | Diasend |
|  | Sugarmate by tandem |
|  | Nightscout |
|  | ShuggahShuggah |
|  | MiniMed mobile and Medtronic carelink |
|  | Diabetes cockpit |
|  | MySugr |
|  | Eversense |
|  | Tandem Control-IQ app |
|  | Juggluco |
|  | Medtronic wecare |
|  | Medtrum easypatch app |
|  | Tidepool Loop app |
|  | AiBetic (Carbohydrate counter) |
| mHealth apps not specific to diabetes but used for carbohydrate counting. | Lifesum |
|  | Livsmedeldatabasen (App giving access to food database released by Livsmedelsverket: the Swedish national food administration) |

^a^This list is based on participant information drawn from quotes. However, it is not exhaustive, as the study did not actively collect data on the specific digital health technology brands used for diabetes self-care.

^b^CGM: continous glucose monitoring

^c^mHealth apps: mobile health applications

### Table S2: mHealth app features used by participants in the survey study (n=143)

| **mHealth app^a^ features used by participants^b^** | **n (%)** |
| --- | --- |
| Automatic data transfer from devices to mHealth app | 137 (95.8) |
| Glucose entry | 136 (95.1) |
| Warning alarm for high or low glucose levels | 130 (90.9) |
| Graphical features | 110 (76.9) |
| Insulin dose registration | 73 (51.0) |
| Reminder | 67 (46.9) |
| Physical activity monitoring | 50 (35.0) |
| Carbohydrate calculator | 47 (32.9) |
| Diet monitoring | 41 (28.7) |
| Contact/share data with healthcare personnel or relatives | 34 (23.8) |
| Insulin bolus calculator | 28 (19.6) |

^a^mHealth apps: mobile health application

^b^Participants have reported use of more than one mHealth app feature. Data missing for n=13 survey participants. The data on mHealth app features used is not available for interview participants.
