# Supplementary material for: Experiences of Adults With Type 1 Diabetes Using Digital Health Technology for Diabetes Self-Care: Qualitative Study
Source: JMIR Diabetes. 2026 Mar 26;11:e79704. doi: 10.2196/79704 (PMC13021107; doi:10.2196/79704)
Supplement: Multimedia Appendix 2 [file diabetes-v11-e79704-s002.docx]

# **Table S3: More example of the data analysis process**

| Meaning Unit | Condensed meaning unit | Code | Subcategory | Category | Theme |
| --- | --- | --- | --- | --- | --- |
| *When I got app on the telephone, I checked my glucose often and in that way took actions on my values in time and in long run got better HbA1c.* [P1, 24 years, male, using CGM and CSII] | Check glucose often taking action on values leading to a better HbA1c. | A better HbA1c from frequent glucose checks and value adjustment. | Aiding glycemic control. | Promoting autonomy in daily life. | A balancing act between feeling empowered and feeling exasperated. |
| *The ability to see over … which times of the day or situations have a greater tendency to end up with high blood sugar. Facilitates making more far-reaching changes beyond the daily decisions you make…. Was able to change the diet in the morning, for example, with good effect.* [P69, 28 years, male, using AID] | Ability to see over time, situations with high glucose and make self-care changes with good effects. | High glucose trend analysis helps making self-care changes | Implementing self-care changes based on value assessment. | Self- awareness through collaborative learning. |  |
| *… the positive thing is that you have control, ….. that nothing will happen in those (occupied) hours or when exercising.....you learn and you get them here….. when you sit in a meeting…. you don't have to think about it...also it is that you have the whole time-24 hour control...* [P454, 48 years, female, using CGM] | It gives control the whole time. Gives access to values when in a meeting or lecturing or exercising and a feeling that nothing will happen during those hours and you do not have to think about diabetes. | DHT giving safety and control the whole time. | Safety and control over the disease. | Feeling secure. |  |
| *When I had the XYZ pump with ABC sensors. The system refused to accept calibrations, thought I had low blood sugar when in fact it was good or high, …therefore (system) stopped the pump and caused high or even higher blood sugar. It was the first times in my life that I experienced ketoacidosis and I have never faced this worse in my entire life. Every day and night was ruined by false alarms, requests for calibration, which were not* *accepted by the system, and incorrect pump stops. I needed to take syringes and run the pump manually or the system would have* *killed me. Obviously, it's very different, which sensors work on which bodies…….ABC sensors have never worked on me, I realized in retrospect. EFG sensors has always worked well for me. …..* [P389, 46 years, male, using AID] | The system refused to accept calibrations. The system incorrectly interpreted low blood sugar, stopped the pump and caused complications. Days and nights were ruined by false alarms, rejected calibrations, and incorrect pump stops. Took syringes and run the pump manually. | Pump malfunction caused complications, need to take syringes and run pump manually. | Hassles with DHT malfunction and usability. | Tackling technical challenges and need for support. |  |
| *Defending beforehand with more/ too much insulin instead of waiting out the curve/ graph for at least two hours. Needs to eat without hunger, broken sleep. Learning: to wait even if difficult with stressful situations to make sure a stable blood sugar curve.* [P7, 58 years, male, using CGM] | Defending beforehand instead of waiting out the graphical curve leads to unwanted consequences. The lesson is to wait out even in stressful situations to make sure a stable blood sugar curve. | Being stressed and defending beforehand (glucose values) leading to insulin overdosing and unwanted consequences. | Psychosocial Hassles related to DHT use. | Navigating the burden of psychosocial challenges. |  |
